# Leaf Surface Topography Contributes to the Ability of *Escherichia coli* on Leafy Greens to Resist Removal by Washing, Escape Disinfection With Chlorine, and Disperse Through Splash

**DOI:** 10.3389/fmicb.2020.01485

**Published:** 2020-07-17

**Authors:** Hung K. Doan, María L. Antequera-Gómez, Atul N. Parikh, Johan H. J. Leveau

**Affiliations:** ^1^Department of Plant Pathology, University of California, Davis, Davis, CA, United States; ^2^Departamento de Microbiología, Instituto de Hortofruticultura Subtropical y Mediterránea “La Mayora”, Universidad de Málaga-Consejo Superior de Investigaciones Científicas (IHSM-UMA-CSIC), Universidad de Málaga, Málaga, Spain; ^3^Department of Biomedical Engineering, University of California, Davis, Davis, CA, United States; ^4^Department of Materials Science and Engineering, University of California, Davis, Davis, CA, United States

**Keywords:** phyllosphere, phyllotelma, PDMS, topomimetic, leaf replicasting, food safety

## Abstract

The attachment of foodborne pathogens to leaf surfaces is a complex process that involves multiple physical, chemical, and biological factors. Here, we report the results from a study designed to specifically determine the contribution of spinach leaf surface topography as it relates to leaf axis (abaxial and adaxial) and leaf age (15, 45, and 75 days old) to the ability of *Escherichia coli* to resist removal by surface wash, to avoid inactivation by chlorine, and to disperse through splash impact. We used fresh spinach leaves, as well as so-called “replicasts” of spinach leaf surfaces in the elastomer polydimethylsiloxane to show that leaf vein density correlated positively with the failure to recover *E. coli* from surfaces, not only using a simple water wash and rinse, but also a more stringent wash protocol involving a detergent. Such failure was more pronounced when *E. coli* was surface-incubated at 24°C compared to 4°C, and in the presence, rather than absence, of nutrients. Leaf venation also contributed to the ability of *E. coli* to survive a 50 ppm available chlorine wash and to laterally disperse by splash impact. Our findings suggest that the topographical properties of the leafy green surface, which vary by leaf age and axis, may need to be taken into consideration when developing prevention or intervention strategies to enhance the microbial safety of leafy greens.

## Introduction

In recent years, fresh produce, in particular leafy greens such as lettuce and spinach, has been recognized as an important vehicle for the transmission of human pathogens that have traditionally been associated with foods of animal origin (Xicohtencatl-Cortes et al., 2009; Scallan et al., 2011). Among these causal agents, enterohemorrhagic *Escherichia coli* O157:H7 is of particular concern as it causes illnesses ranging from diarrhea to life-threatening hemolytic uremic syndrome with as many as 63,000 cases in the United States annually (Scallan et al., 2011).

Contamination of leafy greens by bacterial pathogens of humans is a complex process that is influenced by multiple pre- and post-harvest factors. Some of these factors are well understood, which has spurred the development and implementation of science-based management strategies, such as protocols for produce sanitization, worker hygiene, and manure and irrigation application (Franz and Van Bruggen, 2008; Alegbeleye et al., 2018; Mahajan et al., 2018; Mogren et al., 2018; Riggio et al., 2019).

Other factors that influence pathogen establishment are less well appreciated, which currently prevent them from becoming points-of-departure for the development of practical approaches to pathogen exclusion or removal. One of these factors is the highly heterogeneous nature of the leaf surface, where spatial variation in nutrients (Joyner and Lindow, 2000; Leveau and Lindow, 2001b), water (Axtell and Beattie, 2002), and resident microbiota (Monier and Lindow, 2004) may differentially impact the fate of unwanted enteropathogens as they arrive on and colonize the leaf surface (Monier and Lindow, 2005). This spatial variation is often linked to leaf surface topography: for example, the availability of water and nutrients and the presence of microorganisms is often greater in association with leaf surface features such as veins (Beattie and Lindow, 1995; Beattie, 2011). This makes leaf surface topography a crucial topic of study for understanding the fate of enteropathogens on leafy greens.

To study *E. coli* on leafy greens, whole plants, detached leaves, leaf sections, and even isolated leaf cuticles have been used (Liao and Fett, 2001; Brandl, 2006; Brandl and Amundson, 2008). Studies have started to reveal a role for leaf age and leaf axis in the attachment, persistence, and survival of *E. coli* on leafy greens (Brandl and Amundson, 2008; Van Der Linden et al., 2013). Variation in the establishment and survival of *E. coli* O157:H7 on young vs. old leaves or abaxial vs. adaxial leaf surfaces may be explained by differences in nutrient availability (Brandl and Amundson, 2008; Van Der Linden et al., 2013) and other environmental (Brandl and Mandrell, 2002; Hora et al., 2005; Aruscavage et al., 2008; Lopez-Velasco et al., 2012; Moyne et al., 2013; Van Der Linden et al., 2013; Williams et al., 2013), biological (Tydings et al., 2011; Williams et al., 2013), and physical factors such as leaf roughness or venation (Wang et al., 2009; Macarisin et al., 2013; Hunter et al., 2015). Variation in leaf surface topography, for example between different plant species and cultivars (Tydings et al., 2011; Macarisin et al., 2013), in combination with other conditions such as temperature, relative humidity, and free surface water (Brandl and Mandrell, 2002; Hora et al., 2005; Aruscavage et al., 2008; Tydings et al., 2011) may also underlie the observed variability in effectiveness of chlorine treatments to inactivate *E. coli* (Seo and Frank, 1999; Behrsing et al., 2000; Takeuchi and Frank, 2000; Keskinen et al., 2009).

To deconstruct the complexity and interplay of physical, chemical, and biological processes that occur on leaves, researchers have started to use artificial surfaces that allow for a reductionist approach toward studying the success or failure of *E. coli* to attach, establish, and survive on leafy greens (Zhang et al., 2014; Doan and Leveau, 2015; Doan et al., 2020). Artificial polydimethylsiloxane (PDMS) surfaces consisting of patterned pillars, pits and channels have been used to show how micrometer-scale surface topography affects the dispersal, attachment, resistance to removal, biofilm formation, and survival of *E. coli* (Cao et al., 2006; Mitik-Dineva et al., 2009; Sirinutsomboon et al., 2011; Zhang et al., 2014; Gu et al., 2016, 2017). One disadvantage of such surfaces is that they are relatively poor approximations of the true landscapes of trichomes, stomata, grooves and other features that are present on fresh leaves. Therefore, several research labs have been using fresh leaves as templates in casting protocols to replicate (i.e., topomimetically) the micrometer-scale surface topography of leaves (Sun et al., 2005, 2019; Liu et al., 2007; McDonald, 2013; Zhang et al., 2014; Bernach et al., 2019; Soffe et al., 2019). Using such PDMS leaf replicasts (i.e., reproductions of plant leaf topography in polydimethylsiloxane), Zhang et al. (2014) demonstrated that attachment of *E. coli* cells to grooves between epidermal cells, replicated from PDMS onto agar, better protected the bacteria from biocide treatment than cells growing on flat agar surfaces. In our own work (Doan et al., 2020) we showed that leaf surfaces with greater topography, i.e., more venation, retained more *E. coli* cells than flatter surfaces after brief immersion in a bacterial suspension. Similarly, Sun et al. (2019) demonstrated that retention of spherical colloids on PDMS replicasts of lettuce, spinach, and tomato fruit was dependent on water retention, which was governed by surface roughness and hydrophobicity of the PDMS replica.

The objective of the study presented here was to utilize PDMS spinach leaf replicasts to investigate in more quantitative detail the impact of leaf surface topography as it relates to leaf axis (abaxial and adaxial) and leaf age (15, 45, and 75 day old) on the resistance to removal, escape from chlorine disinfection, and splash dispersal of *E. coli*. Such knowledge is potentially important from a food safety perspective with practical applications such as sanitization protocols and breeding leafy greens for leaf surface topographies that mitigate the attachment, establishment, and survival of *E. coli* on leafy greens.

## Materials and Methods

### Fabrication of PDMS Replicasts

As a source of leaves for the fabrication of PDMS replicasts, we grew *Spinacia oleracea* L. (spinach, variety “Tyee,” up to 75 days) from seed in Sunshine mix #1 (Sun Gro Horticulture, Bellevue, WA, United States) in the greenhouse with 10 h of supplemented light (provided by high-pressure sodium light bulbs), and at temperatures ranging from 27 to 30°C during the day and 18–21°C at night. Using fresh leaves from 15-, 45-, or 75-days-old spinach plants as templates (leaf ages correspond roughly to those of baby spinach, mature spinach, and freezer spinach, see Koike et al., 2011) PDMS leaf replicasts were prepared in a two-step molding process as described in detail previously (Doan et al., 2020). More specifically, the adaxial (top) and abaxial (bottom) sides of a fresh leaf were used to make a negative mold, and each one of those negatives was used to prepare four identical positive PDMS leaf replicasts. From each leaf replicast (representative examples are shown in Supplementary Figure S1), we excised up to 13 circular sections called coupons, using a 24.3 mm inner diameter cork borer: one coupon from 15-days-old leaves; 3–4 coupons from 45-days-old leaves; and 7–13 coupons from 75-days-old leaves. We also prepared flat PDMS replicasts, i.e., lacking any topography, using a glass slide instead of a spinach leaf as template; from these flat PDMS surfaces, coupons were excised in the same way as for leaf replicasts.

### Bacterial Strains and Growth Conditions

*Escherichia coli* ATCC 700728 (*Ec*700728), a non-toxigenic (confirmed lack of Shiga-toxin production genes) BSL-1 rifampicin-resistant derivative of a natural O157:H7 isolate was provided by Dr. Linda Harris (Department of Food Science and Technology, UC Davis) and transformed with plasmid pJBA28 (Andersen et al., 1998) to generate *Ec*700728::JBA28, which carries a chromosomal copy of the *gfp* gene for green fluorescent protein under the control of the P*_*A1/O4/O3*_* promoter and is resistant to kanamycin. *Pantoea agglomerans* 299R::JBA28 (*Pa*299R::JBA28) is a GFP-producing, kanamycin resistant derivative of the rifampicin-resistant phyllosphere model bacterium *P. agglomerans* (formerly known as *Erwinia herbicola*) 299R (Leveau and Lindow, 2001b). *Bacillus velezensis* FZB42 (*Bv*FZB42) was obtained from the Bacillus Genetic Stock Center^1^. All bacterial strains were stored at –80°C in 10% glycerol. In the “Results and Discussion” section, we refer to *Ec*700728::JBA28, *Pa*299R::JBA28, and *Bv*FZB42 as *E. coli*, *P. agglomerans*, and *B. velezensis*, respectively.

Strains *Ec*700728::JBA28 and *Pa*299R::JBA28 were routinely grown at 37° or 28°C on Lysogeny Broth (LB) agar or Tryptic Soy Agar (TSA), respectively, which was supplemented with kanamycin and/or rifampicin to a final concentration of 50 mg per liter. To prepare either strain for inoculation onto PDMS replicasts, a single bacterial colony was transferred from agar medium into LB or into M9 minimal medium supplemented with 0.4% glucose (M9+glucose) (Sambrook et al., 1989) plus appropriate antibiotic(s) and incubated on a rotary shaker, in the dark, at 250 rpm for 10 h at 37°C or 28°C, respectively. Two-hundred microliters of these cultures were transferred into 20 mL of fresh LB or M9+glucose containing Rif_50_ and Kan_50_ and incubated on a rotary shaker (250 rpm) for 6 h at 28°C in the dark to an optical density at 600 nm (OD_600_) of 1–1.5. Bacterial cells were harvested by centrifugation at 2,500 × *g* for 10 min, rinsed twice with sterile water (Milli-Q; Millipore Corporation, Billerica, MA, United States) and resuspended in M9 containing Rif_50_ and Kan_50_ to a final concentration of 10^8^ cells per mL.

As a control for the inoculation of PDMS replicasts with bacterial cells, we prepared a spore suspension of *Bv*FZB42 as follows. A single colony of *Bv*FZB42 was transferred into 100 mL Schaefer’s sporulation medium (Schaeffer et al., 1965) and incubated on a rotatory shaker at 37°C, 200 rpm for 96 h. Endospores were collected by centrifugation at 8,000 rpm for 20 min and resuspended in 100 mL of Tris HCl (50 mM, pH 8.0) amended with 100 μL of a 10 mg/mL lysozyme (Sigma-Aldrich) solution and incubated on a rotary shaker at 200 rpm for 2 h at 37°C to lyse remaining vegetative cells. After centrifugation at 2,500 × g for 10 min, the supernatant was discarded, and the spore pellet was rinsed twice and resuspended in M9 to a final concentration of 10^8^ spores per mL.

As an additional control, we prepared calcofluor-white stained yeast cell wall particles (YCWPblue) of *Saccharomyces cerevisiae* as described previously (Young et al., 2017). They were diluted in M9 to a concentration of 10^8^ particles per mL, as determined by microscopy. We refer to YCWPblue as “yeast particles” in the “Results and Discussion” sections.

### Inoculation and Incubation of Leaf Replicasts

**FIGURE 1:**
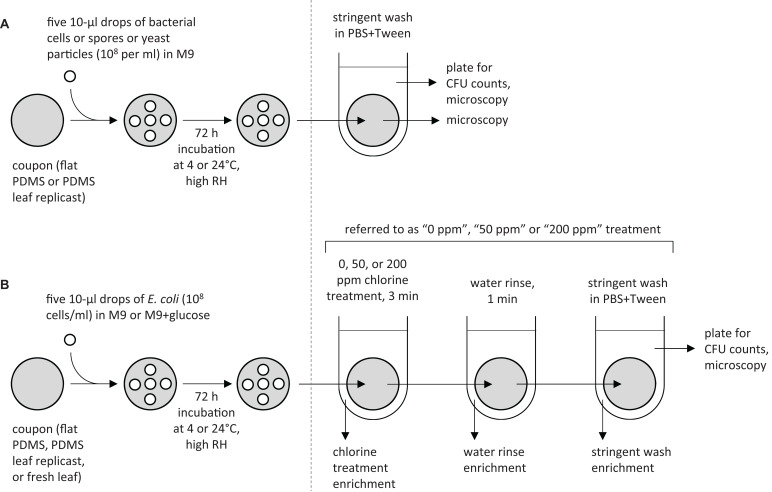
Overview of experiments designed to assess the resistance of *E. coli* and other biomicroparticles to surface removal or chlorine treatment. For the first set of experiments **(A)**, coupons of flat PDMS surfaces or PDMS leaf replicasts (representing different spinach leaf axes and ages) were each inoculated with five 10 μL drops of a 10^8^ per mL suspension of *E. coli* cells, *P. agglomerans* cells, *B. velezensis* spores, or yeast particles in M9 containing the appropriate antibiotic, then incubated and processed as described in detail in “Materials and Methods” section. For the second set of experiments **(B)**, *E. coli* cells were inoculated at 10^8^ cells per mL in M9_Rif50 Kan50_ or M9_Rif50 Kan50_ +glucose onto coupons cut from flat PDMS surfaces, PDMS replicasts of spinach leaves, or fresh spinach leaves, incubated, and processed using “0 ppm,” “50 ppm,” or “200 ppm” chlorine treatments, as described in detail in “Materials and Methods” section. The resulting leaf washes were plated on LB_Rif50 Kan50_ to determine CFUs per coupon. We also performed *E. coli* enrichment protocols immediately after the chlorine wash, water rinse, and stringent wash, as described in the “Materials and Methods” section.

The standard protocol that was used for inoculating PDMS coupons is shown in the left panels of Figures 1A,B. Figure 1A represents protocol A, which was used on coupons cut from flat PDMS surfaces or from PDMS spinach leaf replicasts (adaxial or abaxial side; 15, 45, or 75 days old). Figure 1B represents protocol B, which was used on coupons cut from flat PDMS surfaces, from PDMS replicasts of the abaxial side of 75-days-old spinach leaves, or from fresh spinach leaves (variety “Tyee,” approximately 75 days old; adaxial or abaxial side). In both protocols A and B, inoculation involved pipetting five 10 μL drops on a single coupon in a quincunx pattern. Each drop contained per mL 10^8^ cells (*Ec*700728::JBA28 or *Pa*299R::JBA28), spores (*Bv*FZB42), or yeast particles (YCWPblue) in M9 (protocol A) or 10^8^ *Ec*700728::JBA28 cells in M9 or M9+glucose (protocol B) containing the appropriate antibiotic as described above. Inoculated coupons were placed in a parafilmed 100 × 15 mm diameter plastic Petri dish (VWR International) with one layer of germination paper (catalog number CDB 3 3/8″ Circle; Anchor Paper Co., Saint Paul, MN, United States) that was saturated with 4.5 mL of sterile deionized water (to create wat we refer to here as a high relative humidity). Petri dishes were incubated at 4 or 24°C: these temperatures are typical during harvest (24°C), processing (24°C/4°C), or storage (4°C) of leafy greens (Beuchat and Ryu, 1997; U.S. Food Drug Administration, 2010).

### Processing of Leaf Replicasts

After 72 h incubation, coupons were processed as depicted in the right panels of Figures 1A,B. For protocol A, coupons were individually transferred into 5 mL of sterile phosphate buffer solution (PBS, 10 mM, pH 7.2) amended with 0.2% Tween 20 (Thermo Fisher Scientific, Waltham, MA, United States), vortexed vigorously for 30 sec, sonicated in a sonicator bath with adjustable power (Branson Ultrasonics Corp, St. Louis, MO, United States) at 250 W (frequency 40 kHz) for 5 min, and vortexed briefly again for 15 s. Dilutions of the resulting “stringent wash” were spread-plated in triplicate on LB Rif_50_ Kan_50_ (*Ec*700728::JBA28) or 0.1x TSA Rif_50_ Kan_50_ or without antibiotic (*Pa*299R::JBA28, *Bv*FZB42, respectively) using an Eddy Jet spiral plater (IUL, Barcelona, Spain). Surface washes containing yeast particles were not plated, but instead concentrated by centrifugation at 2500 × *g* for 10 min, resuspended in 1 mL of sterile water and enumerated with a hemocytometer. For protocol B, coupons were submerged in 10 mL of 0, 50, or 200 ppm available chlorine in water (PrimeSource Germicidal Ultra Bleach, Reliable Redistribution Resource, Fremont, CA, United States) for 3 min (“chlorine treatment”), followed by submersion in 20 mL of sterile water for 1 min (“rinse”), and transfer into 5 mL of PBS-Tween. Each coupon was then processed as above, resulting in a “stringent wash” solution that was dilution-plated in triplicate on LB agar containing Rif_50_ and Kan_50_.

For both protocols A and B, the counts of colony-forming units (CFUs) or yeast particles recovered in the stringent wash from each coupon were ^10^log-transformed, after replacing all zero values with LOD/2, where LOD is the limit of detection (Croghan and Egeghy, 2003) then averaged over the triplicate agar plates per coupon. The CFU count of each coupon was plotted as a function of the percentage of surface venation on that same coupon, as determined by microscopy using ImageJ^2^. We also averaged the count of bacterial cells/spores or yeast particles for all coupons cut from the same leaf, to obtain an average count per coupon for each leaf, of which there were three replicates for each type of leaf surfaces (i.e., 15, 45, or 75 days old, and abaxial or adaxial) plus three replicates of a flat surface.

In both protocols A and B, stringent wash solutions and the stringently washed coupons were examined for (residual) bacterial cells or spores or yeast particles by fluorescence microscopy using an Axio Imager M2 microscope (Zeiss, Jena, Germany). Furthermore, in protocol A, the surfaces of washed coupons were wiped with a sterile dampened cotton swab that was then streaked along the surface of LB agar containing Rif_50_ and Kan_50_ (*Ec*700728::JBA28) or 0.1x TSA containing Rif_50_ and Kan_50_ or without antibiotic (*Pa*299R::JBA28, *Bv*FZB42, respectively) to test for bacterial growth. In protocol B, we also checked each step in the protocol (i.e., “chlorine treatment,” “water rinse,” and “stringent wash”) for viable *E. coli* by adding 1/10th-volume of 10x LB containing Rif_50__0_ and Kan_50__0_, followed by incubation at 37°C for 24 h to enrich for *E. coli* growth. Samples were scored positive or negative for bacterial growth; positive growth was confirmed to be *E. coli* using an Axio Imager M2 fluorescence microscope (Zeiss).

### Dispersal of *E. coli* on Leaf Surfaces by Splash

To determine how leaf topography affected the short-distance, lateral dispersal of *E. coli* by splash, we placed single coupons from the abaxial side of 75-days-old fresh spinach leaves, from replicasts representing the abaxial side of 75-days-old fresh spinach leaves, or from flat replicasts, in the center of an LB Rif_50_ agar plate (100 × 15 mm diameter plastic Petri dish). A single 10-μL drop with 10^6^ CFU of *Ec*700728 per ml was deposited onto the center of each coupon surface to create a source point. Immediately after, one 30-μL sterile deionized water droplet was dropped from a height of 30 cm onto the center of the coupon surface. Coupons were carefully removed from the agar surface, and the plates were incubated at 37°C for 24 h. For each plate, we recorded the number of colonies and the distance of each colony from the source point. For each coupon, the splash experiment was repeated two times, for a total of three replications per coupon. We calculated the average number of *E. coli* colonies and the average dispersal distance for three replicated experiments. From these data, we also averaged the dispersal distance of *E. coli* colonies for all coupons cut from the same leaf, to obtain an average count per coupon for each leaf, of which there were three different sets of leaves per experiment, with each set containing four identical PDMS flat/leaf replicasts or four fresh leaves each with 7–13 coupons.

### Data Analysis

The data were analyzed using SAS version 9.4 (SAS Institute, Inc. Cary, NC, United States) or using R version 3.3.2 (The R Foundation for Statistical Computing, Vienna, Austria) with a general linear mixed effects model and analysis of variance (ANOVA) for least significant differences among the combinations of treatments (*P*< 0.05). Means were separated by Tukey’s HSD at *P* <0.05.

## Results

### Recovery of *E. coli* as a Function of Leaf Topography, Axis, and Age

**FIGURE 2:**
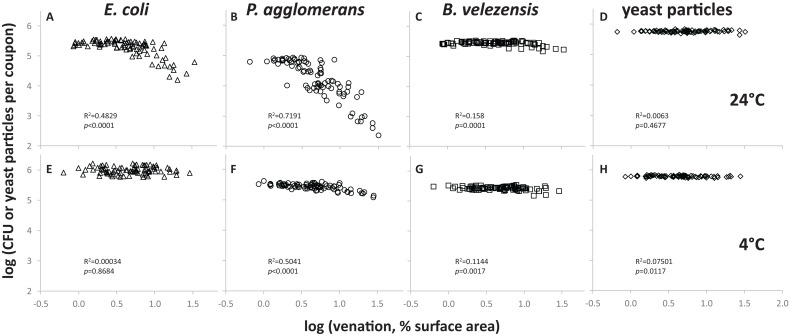
Surface topography-dependent resistance of *E. coli* to removal from PDMS leaf replicasts by stringent washing. Each data point represents a single coupon that was inoculated with *E. coli* cells **(A,E)**, *P. agglomerans* cells **(B,F)**, *B. velezensis* spores **(C,G)** or yeast particles **(D,H)**, then incubated at 24°C **(A–D)** or 4°C **(E–H)**, and washed as depicted in Figure 1A. For each coupon, the count of bacterial CFUs (*E. coli*, *P. agglomerans*, *B. velezensis*) or yeast particles in the wash was plotted as a function of venation, i.e., the percentage of surface area covered by veins. Shown in each panel are between 84 and 88 coupons, representing PDMS leaf replicasts from the adaxial or abaxial surfaces of 15-, 45-, or 75-days-old spinach leaves, with three leaves per age per axis and between 1 (15-days-old plants) and 13 (75-days-old plants) coupons per leaf. In each panel, goodness of fit of the data points to a simple regression line (R^2^) and *p*-values (is the slope of that line significantly different from zero?) are provided. These were calculated using a linear regression calculator (GraphPad).

To quantify the impact of leaf surface topography on *E. coli*’s resistance to removal, we spot-inoculated coupons cut from PDMS replicasts of spinach leaves representing different leaf axes and ages, with five 10 μL drops of a 10^8^ cell/mL suspension in M9, incubated the coupons at 24°C for 72 h at high relative humidity (as defined in “Materials and Methods” section), and then measured by spread-plating the number of CFUs that could be retrieved from the surface using a stringent wash protocol (Figure 1A). When these CFU counts were plotted for each coupon as a function of the percentage of surface covered by leaf veins, the recovery of *E. coli* cells was found to be lower from coupons with greatest venation (Figure 2A). An even more pronounced topography-dependent resistance to removal was observed with cells of a phyllosphere isolate of *P. agglomerans*, used here as a control (Figure 2B). When coupons inoculated with *E. coli* or *P. agglomerans* cells were incubated at 4°C instead of 24°C, the size of the effect of venation on recovery was diminished (Figures 2E,F, respectively). The impact of venation on recovery of two other controls, i.e., spores of *B. velezensis* (Figures 2C,G) and yeast particles (Figures 2D,H), either at 24°C (Figures 2C,D) or 4°C (Figures 2G,H), was minimal or non-significant.

Using fluorescence microscopy, we observed significant numbers of residual cells of *E. coli* and *P. agglomerans* on coupons from which fewer CFUs were recovered following stringent washes (Supplementary Figure S2). These bacterial cells were green fluorescent, indicating that they were viable. Many cells were found associated with leaf veins. Also, after surface-swabbing these same coupons and streaking the swab on agar medium, we observed colonies (not shown) suggesting that the bacteria that were protected from removal by stringent wash were viable and removable by physical force. No bacterial cells were observed on stringently washed coupons that were incubated at 4°C instead of 24°C.

**FIGURE 3:**
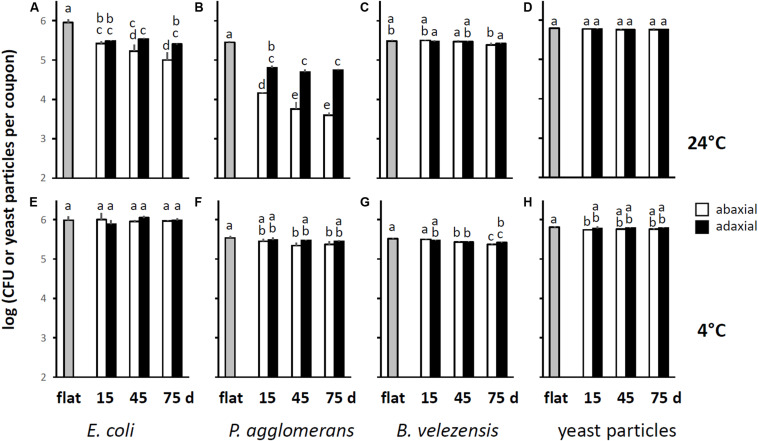
Resistance of *E. coli* and *P. agglomerans* cells, *B. velezensis* spores, and yeast particles to removal by stringent washing from PDMS replicasts as a function of leaf axis and age, and temperature. These graphs were generated using the data shown in Figure 2, by averaging the counts of CFUs retrieved from coupons representing PDMS replicasts of the adaxial or abaxial side of three independent leaves from either 15-, 45-, or 75-days-old plants. Shown are the means and standard deviations across these three leaves. **(A–D)** Show data from coupons incubated at 24°C, **(E–H)** from coupons incubated at 4°C. White bars represent abaxial surfaces, black bars adaxial surfaces. Gray bars show the means and standard deviations for three sets of 4 coupons each cut from flat PDMS surfaces. Within each panel, bars with the same letter represent values that were not significantly different from each other.

When the CFU data were averaged by leaf axis and age, it became evident that after incubation at 24°C, *E. coli* cells were significantly more difficult to retrieve from PDMS replicasts of abaxial compared to adaxial leaf surfaces of 75-days-old spinach (Figure 3A). Such differences between abaxis and adaxis were not significant for younger leaves (Figure 3A). However, PDMS replicasts of both axes at all three plant developmental stages (15-, 45-, and 75-days-old) showed a significantly lower recovery rate than flat PDMS surfaces (Figure 3A). By contrast, we observed no significant differences in the retrieval of *E. coli* cells between flat PDMS surfaces and PDMS leaf replicasts when coupons were inoculated at 4°C (Figure 3E). Also, the numbers of *E. coli* cells that could be retrieved from flat surfaces after incubation at 4°C were not significantly different from the numbers of *E. coli* cells that could be retrieved from flat surfaces after incubation at 24°C (*p* = 0.880). This suggests that temperature did not impact the ability of *E. coli* cells to attach to PDMS.

Cells of *P. agglomerans* were significantly more difficult to retrieve, but only after incubation at 24°C, from (1) abaxial surfaces compared to adaxial surfaces (independent of leaf age), (2) the abaxial surfaces of 45- and 75-days-old leaves compared to 15-days-old leaves, and (3) any type of PDMS leaf replicast compared to flat PDMS surfaces (Figure 3B). At 4°C, retrieval was not significantly different between flat and adaxial surfaces, or between abaxial and adaxial surfaces (Figure 3F). For the other two controls (*B. velezensis* spores and yeast particles), differences in retrieval between flat and leaf-mimetic surfaces were either insignificant or significant but relatively small compared to what was observed for *E. coli* and *P. agglomerans*, both at 24° (Figures 3C,D, respectively) and 4°C (Figures 3G,H, respectively).

### Influence of Leaf Surface Topography on Resistance to Chlorine Treatment of *E. coli*

To assess the effect of leaf surface topography on the resistance of *E. coli* to foliar treatment with chlorine, we inoculated coupons cut from flat PDMS surfaces or from PDMS replicasts of the abaxial side of 75-days-old spinach leaves with five 10 μL drops of a 10^8^ CFU per mL suspension of *E. coli* in M9 and then incubated these coupons at 4° or 24°C for 72 h at high relative humidity (as defined in “Materials and Methods” section), as described above. Instead of subjecting these coupons directly to a stringent wash protocol, we first immersed them in a solution of 0, 50, or 200 ppm available chlorine in water for 3 min, rinsed them in water for 1 min, and then measured by spread-plating the number of residual CFUs that could be retrieved from the surface using the stringent wash protocol (Figure 1B). We refer to these series of washes (3 min in 0, 50, or 200 ppm available chlorine, then 1 min water rinse, then a stringent wash) as the “0 ppm,” “50 ppm,” or 200 ppm” treatment, respectively.

**FIGURE 4:**
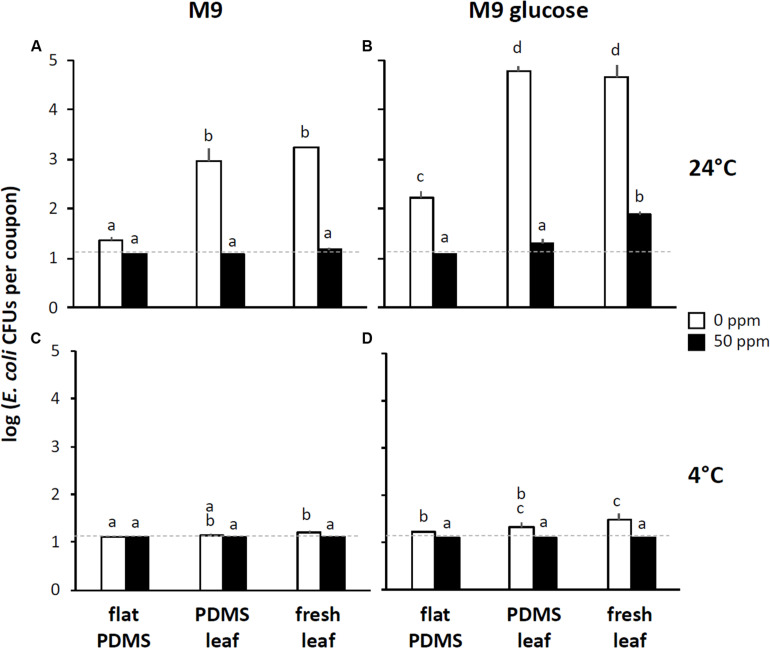
Impact of surface topography, nutrient availability, temperature, and chlorine washes on the recovery of *E. coli* from leaf surfaces. For these experiments, coupons of flat PDMS surfaces (“PDMS flat”), PDMS replicasts of the abaxial side of 75-days-old spinach leaves (“PDMS leaf”), or abaxial side of 75-days-old fresh spinach leaves (“fresh leaf”) were inoculated with *E. coli* in M9 (left panel) or M9+glucose (right panel), incubated, chlorine-treated, rinsed, and washed as depicted in Figure 1B. For each one of three independent leaves (PDMS or fresh) or three flat PDMS surfaces, the values of CFUs per coupon were averaged, and shown here are the means and standard deviations of coupons across these three leaves or flat surfaces. Black bars represent means from the “50 ppm” treatments, white bars from the “0 ppm” treatments. **(A,B)** Show data from coupons incubated at 24°C, **(C,D)** from coupons incubated at 4°C. Not shown are data for the “200 ppm” treatments (for which all values were below the limit of detection, which in these graphs is indicated by the stippled line). Within each panel, bars with the same letter represent values that are not significantly different from each other.

**FIGURE 5:**
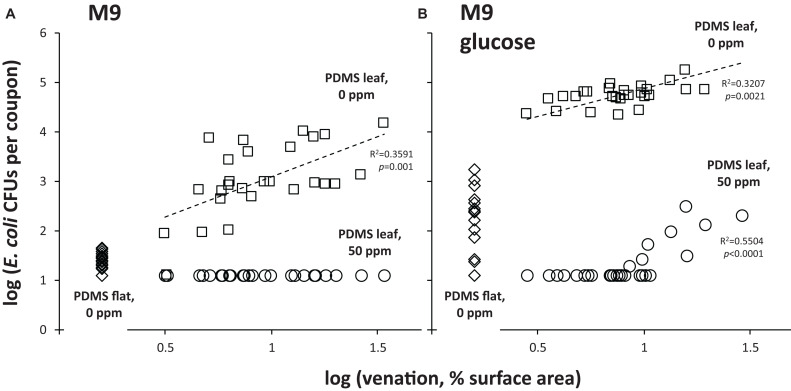
Resistance of *E. coli* to chlorine and removal from PDMS surfaces as a function of leaf venation and nutrient availability. Shown are retrievable CFU data as a function of leaf venation for individual coupons from PDMS leaf replicast coupons that were subjected to the “0 ppm” (circles) or “50 ppm” (squares) treatment, as well as from flat PDMS surface coupons subjected to the “0 ppm” treatment (diamonds). Not shown are the data for the “50 ppm”-treated flat PDMS surface coupons (all below limit of detection). **(A)** Shows data from coupons that were inoculated with *E. coli* in M9, **(B)** from coupons inoculated with *E. coli* in M9+glucose. Shown are goodness of fit of data points to a simple regression line (R^2^) and *p*-values (is the slope of that line significantly different from zero?). These were calculated using a linear regression calculator (GraphPad).

The baseline for this experiment was the “0 ppm” treatment: it showed how many viable *E. coli* could still be retrieved from PDMS surfaces by stringent washing after first immersing and rinsing the same surfaces in water (i.e., a non-stringent wash). For flat PDMS surfaces incubated at 24°C, CFU counts were close to the limit of detection (about 10 CFUs per coupon), and significantly lower (about 2 orders of magnitude) than the counts of CFUs that were retrieved from PDMS leaf replicasts (Figure 4A). This suggests a topography-dependent resistance to surface removal by non-stringent immersion and rinse in water. In support of this notion, we observed a positive relationship between the level of venation of individual coupons cut from PDMS leaf replicasts and the number of CFUs that were retrieved from those same coupons (Figure 5A, squares).

When the same experiment was done with the “50 ppm” treatment, counts of retrievable *E. coli* CFUs were below the limit of detection, both for flat PDMS and PDMS leaf replicasts (Figure 4A), and independent of the degree of leaf venation (Figure 5A; circles). For flat surfaces, the impact of the “50 ppm” treatment was not significantly different from washing without chlorine (Figure 4A). However, with PDMS leaf replicasts, the same “50 ppm” treatment reduced *E. coli* counts significantly by two orders of magnitude on average (Figure 4A).

For comparison, we also performed the “0 ppm” and “50 ppm” treatments with coupons cut from the abaxial side of fresh leaves from 75-days-old spinach plants. The results were not significantly different from those obtained with PDMS leaf replicasts, although the average CFU count after the “50 ppm” treatment was slightly above the limit of detection (Figure 4A).

One important way in which fresh leaves and PDMS leaf replicasts differ is that the surfaces of the former harbor nutrients such as sugars, which can be used by *E. coli* and other bacteria to multiply. This led us to test the topography-dependent resistance of *E. coli* to surface removal and chlorine treatment under conditions of nutrient availability by inoculating coupons of flat PDMS and PDMS leaf replicasts with *E. coli* cells in glucose-supplemented M9. The availability of nutrients during the incubation period at 24°C increased the counts of retrievable CFUs from both flat and leaf PDMS surfaces by immersion and rinse in water (the “0 ppm” treatment) as would be expected. Importantly, however, as was observed in the absence of glucose (Figure 4A), there was a significant difference (about 2.5 orders of magnitude) in CFU counts retrieved between flat PDMS and PDMS leaf replicasts in the presence of glucose (Figure 4B). Consistent with this, we found a similar positive correlation with leaf venation (Figure 5B, squares).

**FIGURE 6:**
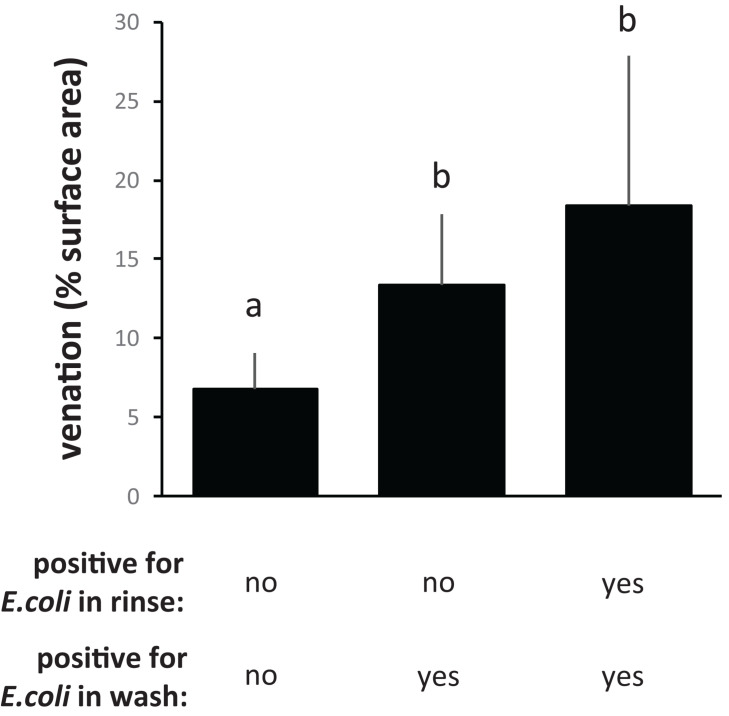
Topography-dependent enrichment of *E. coli* in water rinses and stringent washes, following treatment with 50 ppm available chlorine. Shown are the averages and standard deviations of percentages of leaf surface venation for PDMS leaf replicast coupons shown in Figure 1B (circles) which scored negative for *E. coli* both in the water rinse and stringent wash, coupons that scored negative for *E. coli* in the rinse wash but positive in the stringent wash, and coupons that scored positive for *E. coli* both in the water rinse and stringent wash. Bars with the same letter represent values that were not significantly different from each other.

With the “50 ppm” treatment, CFU counts from flat surfaces dropped about 10-fold compared to the “0 ppm” treatment, to below the limit of detection, whereas CFU counts from PDMS leaf replicast surfaces and fresh leaves dropped as much as 1,000–10,000-fold to an average just above the limit of detection (Figure 4B). The few PDMS leaf replicast coupons from which CFUs could be retrieved were coupons with a relatively high percentage of surface area covered by veins (Figure 5B, circles). To further assess the contribution of leaf surface venation on the resistance of *E. coli* to the “50 ppm” treatment, we subjected the “chlorine immersion,” “water rinse” and “stringent wash” solutions for each coupon (Figure 1B) to an *E. coli* enrichment protocol and scored each coupon as either positive or negative for *E. coli*. While all “chlorine immersion” solutions scored negative for *E. coli* (not shown), those coupons that scored negative for *E. coli* both in the “water rinse” and “stringent wash” solutions showed a significantly lower degree of venation on average than coupons that scored (1) negative for *E. coli* in the “water rinse” but positive in the “stringent wash” solution, or (2) positive for *E. coli* both in the “water rinse” and “stringent wash” solutions (Figure 6). These findings are consistent with the notion that leaf surface topography, i.e., venation, facilitates the escape of some *E. coli* cells from the “50 ppm” treatment.

When the “0 ppm” and “50 ppm” treatments were repeated at 4°C instead of 24°C, the difference in recovery of *E. coli* from flat PDMS and PDMS leaf replicast surfaces was much smaller overall, both in the absence (Figure 4C) and presence (Figure 4D) of glucose during incubation on the coupons. This confirms a role of temperature in the resistance of *E. coli* to removal from PDMS leaf replicasts. With the “200 ppm” treatment, we never recovered viable *E. coli* from any coupons (whether cut from PDMS surfaces or from fresh leaves), with or without glucose or whether at 4° or 24°C. This result highlights the efficacy of a surface wash with 200 ppm available chlorine to inactivate *E. coli*.

### Topography-Dependent Dispersal of *E. coli* From Leaf Surfaces by Water Splash

**FIGURE 7:**
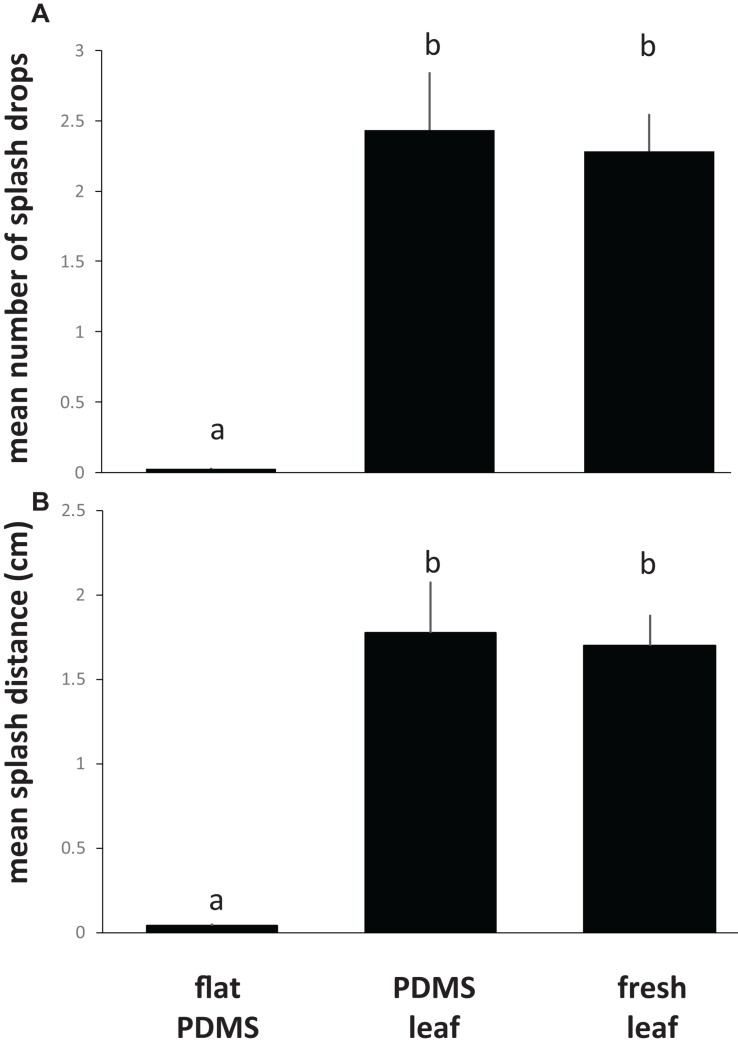
Impact of leaf surface topography on the short-distance, lateral dispersal of *E. coli* through splash impact. For this experiment, *E. coli* cells were inoculated in the center of coupons cut from flat PDMS surfaces, from PDMS replicasts of the abaxial side of leaves from 75-days-old spinach plants, or the abaxial side of fresh leaves from 75-days-old spinach plants. Coupons were placed in the center of an LB agar plate, and a 30 μL drop of water was released from a height of 30 cm above the center of the coupon. Coupons were removed from the agar and the plates were incubated at 37°C to determine the amount of splash created by the impact of the drop **(A)** or the mean distance of splash drops from the center of the coupon **(B)**. Shown are the average and standard deviations for coupons from three independent leaves (PDMS or fresh) or flat surfaces. Bars with the same letter represent values that are not significantly different from each other.

**FIGURE 8:**
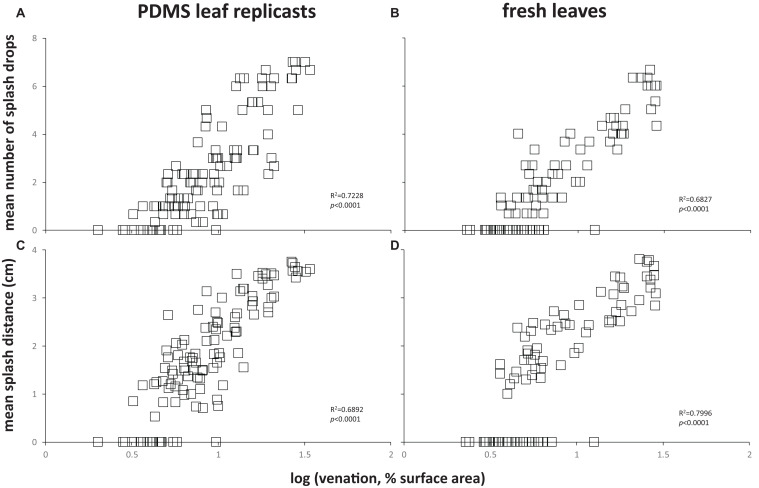
Correlation between leaf venation and lateral dispersal of *E. coli* through splash impact. Each data point represents the amount of splash created by the impact of the drop **(A,B)** or the mean distance of splash drops from the center of the coupon **(C,D)** for a single coupon. These are the same data as shown in Figure 7 for PDMS leaf replicasts or fresh leaves. In each panel, goodness of fit of the data points to a simple regression line (R^2^) and *p*-values (is the slope of that line significantly different from zero?) are provided. These were calculated using a linear regression calculator (GraphPad).

To assess the effect of leaf topography on short-distance, lateral dispersal of *E. coli* by water splash, we spot-inoculated the center of coupons cut from flat PDMS surfaces, PDMS leaf replicasts (abaxial, spinach, 75 days old), or fresh leaves (abaxial, spinach, 75 days old) with a single, 10 μL drop of 10^6^ cells of *E. coli* per mL, placed the inoculated coupon on an LB agar surface, then dropped a 30 μL drop of sterile water from a height of 30 cm onto the center of the coupon surface. Coupons were carefully removed from the agar surface, and plates were incubated to allow growth of *E. coli*. We measured the number of CFUs (equal to the number of splash drops) as well as the average distance of splash drops away from the center of the coupon. These measurements revealed a significant impact of leaf surface topography (whether on PDMS leaf replicasts or fresh leaves) on both the number of splash drops (Figure 7A) and the splash distance (Figure 7B) compared to flat PDMS surfaces. When broken down by individual coupons, there was a clear correlation between the percentage of surface covered by venation and the number of splash drops generated (Figures 8A,B) or the splash distance (Figures 8C,D). This was true both for PDMS leaf replicasts (Figures 8A,C) and fresh leaves (Figures 8B,D).

## Discussion

The establishment and survival of foodborne pathogens such as *E. coli* O157:H7 on leafy greens are complex processes that involve interactions between many physical, chemical, and biological factors (Brandl and Amundson, 2008; Tydings et al., 2011; Macarisin et al., 2013; Van Der Linden et al., 2013). To deconstruct some of this complexity, PDMS replicasts of abaxial and adaxial surfaces of 15-, 45-, and 75-days-old spinach leaves were used in this study to quantitatively investigate the role of leaf surface topography on the resistance to removal, survival, and dispersal of *E. coli* on leafy greens. Leaves at different developmental stages were chosen for this study because fresh spinach is consumed at these developmental stages and leaf surface topographies are noticeably different at these stages. Under standardized conditions (i.e., incubated in the absence of nutrients and at 24°C), bacterial resistance to removal was a function of leaf topography, where the recovery of *E. coli* cells was lower from coupons with greater venation (Figures 2A, 5). Cells were significantly more difficult to recover from abaxial surfaces compared to adaxial surfaces, and from the abaxial surfaces of 75-days-old leaves compared to 15-days-old leaves (Figure 3A). Also, more cells of *E. coli* could be retrieved with stringent washing from not-stringently washed leaf topomimetic surfaces than with stringent washing from not-stringently washed flat surfaces (Figure 4A). Topography-dependent resistance to removal of *E. coli* is consistent with observations reported by Macarisin et al. (2013) using fresh spinach leaves. Similarly, Wang et al. (2009) found a positive correlation between surface topography of fruit surfaces and adhesion rate of *E. coli* (Wang et al., 2009). Hunter et al. (2015) reported that *Salmonella enterica* was more difficult to remove from older lettuce leaves than younger ones and that these differences were associated not only with leaf venation but stomatal densities, which for some (but not all) spinach varieties significantly differs between abaxial and adaxial surfaces (Lopez-Velasco et al., 2011). Important to note is that the above mentioned studies were done with fresh leaves, which makes it difficult to separate the contribution of leaf surface topography from other factors, such as leaf surface chemicals, hydrophobicity, and resident microbiota. Our experiments with PDMS leaf replicasts allow us to rule out such factors and to establish a contribution of leaf topography, in particular venation.

One possible explanation for the topography-dependent resistance to removal is the increased surface area that is available for adherence of *E. coli* cells, or of water bodies containing *E. coli* cells, to topomimetic PDMS surfaces compared to flat PDMS surfaces. Such increased surface area has been proposed as an explanation for the difficulty of removing *E. coli* from rough surfaces (Wang et al., 2009; Macarisin et al., 2013; Hunter et al., 2015). Under the experimental conditions tested here, very few bacteria remained attached to flat PDMS surfaces after stringent washes (as observed by microscopy) suggesting that direct attachment to PDMS may not be a major factor in the observed resistance to removal. A more likely explanation is that *E. coli* cells got confined in spaces created by leaf surface features such as veins and maybe stomata, or within bodies of water associated with those leaf features such that they were protected from forces aimed to remove them during the stringent washing protocol (Characklis, 1981; Wang et al., 2009; Hunter et al., 2015). In our study, this is evidenced by the observation of residual bacterial cells near leaf venation with fluorescence microscopy after leaf washing (Supplementary Figures S2B,D). Such protection is absent from flat PDMS surfaces.

Chlorine is widely used by the leafy greens industry because of its ability, at low cost, to inactivate foodborne pathogens, and its minimal impact on the nutritional and aesthetic quality of produce. The United States Food and Drug Administration recommends 50–200 ppm of available chlorine at pH 6.0–7.5 and contact times of 1–2 min (U.S. Food Drug Administration, 1998). Many studies have revealed a broad range of chlorine efficacy in inactivation of *E. coli* on leafy greens (Seo and Frank, 1999; Behrsing et al., 2000; Takeuchi and Frank, 2000; Keskinen et al., 2009). Variation in efficacy may be attributed to differences in a number of factors, including leaf damage, internalization of *E. coli* cells, and the inactivation of chlorine by organic material associated with lettuce leaves (Seo and Frank, 1999; Behrsing et al., 2000; Takeuchi and Frank, 2000; Keskinen et al., 2009). The results we show here suggest that variation in leaf surface topography is another explanatory factor for differences in survival of *E. coli* on chlorine-treated leafy greens (Figures 5, 6). Surface roughness of plant leaves has been implemented as a factor protecting *E. coli* cells from treatment with chlorine (Wang et al., 2009; Fransisca and Feng, 2012). This is also consistent with work by Zhang et al. (2014) who demonstrated that attachment of *E. coli* cells to grooves between epidermal cells, replicated from PDMS onto nutrient agar, better protected the bacteria from chlorine treatment at 200 ppm than cells growing on flat agar surfaces. Takeuchi and Frank (2000), similarly, found that lettuce leaf structures played an important role in the protection of *E. coli* O157:H7 cells from chlorine inactivation, as cells located near depressions in the cuticle survived chlorine treatments. Combined, these observations are consistent with a model in which leaf topography protects *E. coli* cells on the surface directly from getting into contact with chlorine and/or protects *E. coli* cells from being removed from the surface and indirectly from inactivation by chlorine in the wash water.

A topography-dependent resistance to removal was also observed with cells of *P. agglomerans* (Figure 2B). Cells of *P. agglomerans* were significantly harder to retrieve from the abaxial surface compared to adaxial surfaces, and from the abaxial surfaces of 45- and 75-days-old leaves compared to 15-days-old leaves (Figure 3B). The strain of *P. agglomerans* that we used in our study was originally isolated from plant foliage and is expected to be a better colonizer of plant surfaces than foodborne pathogens (Takeuchi et al., 2000; Brandl and Mandrell, 2002; Garrood et al., 2004; Brandl, 2006). We suspect that *P. agglomerans* has evolved features that maximize its retention on leaf surfaces and that such features are absent in less-phyllosphere-fit bacteria such as *E. coli*. No effect of topography was observed on the recovery of spores of *B. velezensis* (Figures 2C,G) and yeast particles (Figures 2D,H). A possible explanation for this observation lies in how bacterial spores and yeast particles differ in size and surface characteristics from bacterial cells, which is likely to differentially impact their interaction with leaf topographies and its associated water landscape.

In our experiments, the topography-dependent resistance to removal of *E. coli* or *P. agglomerans* cells was more pronounced when coupons were incubated at 24°C than at 4°C (Figures 2E,F, respectively). Many studies have reported the importance of temperature on the surface attachment of both foodborne pathogens and phyllosphere epiphytes (Maurelli et al., 1984; Brandl and Mandrell, 2002; Allwood et al., 2004; Barnhart and Chapman, 2006; Ells and Truelstrup Hansen, 2006; Xicohtencatl-Cortes et al., 2009; Dourou et al., 2011; Van Der Linden et al., 2014). However, seeing that in our experimental setup, there was no significant difference in *E. coli* removal from flat PDMS coupons that were incubated either at 4°C or at 24°C, the differences that we observed with leaf topomimetic PDMS surfaces at these two temperatures are most likely not due to differential attachment of *E. coli* cells to the PDMS surface. More likely, the effect of temperature is linked to interactions between the PDMS surface and the water drops, and/or interactions between the bacteria and the water drops. Perhaps temperature impacts the ability of bacteria to swim in the water landscape and to explore and end up in crevices and other features of the leaf topography that offer protection from later being washed off. This would be consistent with our observation that the effect of temperature was much less obvious or non-existent for *Bacillus* spores (Figures 3C,G) and yeast bioparticles (Figures 3D,H), neither of which possess the ability to swim.

Leaves harbor nutrients such as sugars that can be utilized by bacteria (Tukey and Tukey, 1966; Mercier and Lindow, 2000; Leveau and Lindow, 2001a; Brandl, 2006). *E. coli* O157:H7 can grow on several nutrients found on sliced cucumbers (Abdul-Raouf et al., 1993) shredded spinach and lettuce (Abdul-Raouf et al., 1993; Diaz and Hotchkiss, 1996; Cooley et al., 2006) and cantaloupe and watermelon cubes (Del Rosario and Beuchat, 1995) and in apple juice (Zhao et al., 1993). Nutrients have been shown to be a major factor in the attachment of *E. coli* to surfaces and its survival in the face of biocidal treatments (Herson et al., 1987; Beuchat, 1999; Li et al., 2001; Hassan and Frank, 2004; Ryu et al., 2004). In our experiments, glucose was used, which is one of the predominant sugars found on leaf surfaces (Mercier and Lindow, 2000; Cooley et al., 2006). Our findings support the notion that nutrient availability stacks the odds in favor of *E. coli* by giving it an opportunity to multiply, which increases its chances of persisting on leaf surfaces when challenged with a chlorine wash (Figure 5B).

It has been demonstrated that foliar irrigation can act as a vector for *E. coli* from soil (Mootian et al., 2009; Monaghan and Hutchison, 2012) fecal matter (Mootian et al., 2009; Atwill et al., 2015) or furrow water splash onto leafy greens (Mootian et al., 2009; Monaghan and Hutchison, 2012; Atwill et al., 2015). To the best of our knowledge, no studies have so far looked at the dispersal of *E. coli* by splash to other parts of the same leaf or to other nearby leaves of crops such as lettuce and spinach. In our study, we demonstrated a significant impact of leaf surface topography on the dispersal of *E. coli* by water splash, showing a higher number of splash drops as well as further splash distance from coupons with greater surface leaf topography, i.e., more venation. Our study suggests that surface topography may impact splash-driven cross-contamination of *E. coli* in pre- and post-harvest and food-processing environments. The effect of surface roughness on splash dispersal has been previously reported with fungal spores on leaves (Fitt and Lysandrou, 1984; Madden et al., 1996) fruit surfaces (Grove, 1985; Reynolds, 1989; Madden et al., 1996) and artificial leaf surfaces (Hörberg, 2002).

In summary, using experimentally amenable PDMS spinach leaf replicasts as a model surface, we were able to show that the resistance to removal, survival, and dispersal of *E. coli* O157:H7 on spinach is significantly affected by leaf surface topography (which varies by leaf axis and leaf age) in combination with temperature and nutrients. Leaf surface topography also was a contributing factor in the foliar survival of *E. coli* following chlorine treatment, with significantly greater numbers of viable cells on surfaces with more pronounced leaf venation (i.e., greater leaf roughness). Surface topography, as it relates to leaf age, may need to be taken into consideration by breeders and growers in selecting fresh-market spinach cultivars.

## Data Availability Statement

The raw data supporting the conclusions of this article will be made available by the authors, without undue reservation, to any qualified researcher.

## Author Contributions

JL secured funding for this study. HD, JL, and AP did the experimental design. HD and MA-G performed the experiments. HD and JL wrote the draft of the manuscript with editing of relevant sections by other authors. All authors contributed to the article and approved the submitted version.

## Conflict of Interest

The authors declare that the research was conducted in the absence of any commercial or financial relationships that could be construed as a potential conflict of interest.

## Footnotes

1 http://www.bgsc.org

2 http://rsb.info.nih.gov/ij/

## Supplementary Material

FIGURE S1 Representative PDMS replicasts of abaxial and adaxial surfaces of spinach leaves of different ages. Each one of the replicasts is shown with the coupons that were cut from it using a 24.3 mm diameter corkborer (for the purpose of this figure, coupons were placed back into the cut-out).

FIGURE S2 Fluorescence microscopy images of residual bacterial cells of *E. coli* O157:H7 **(A,B)** and *P. agglomerans* cells **(C,D)** on PDMS spinach leaf replicasts after coupons were washed with 5 mL of sterile PBS amended with 0.2% Tween 20, vortexed vigorously for 30 s and then sonicated.
