# Supplementary figures and images for: Leaf Surface Topography Contributes to the Ability of Escherichia coli on Leafy Greens to Resist Removal by Washing, Escape Disinfection With Chlorine, and Disperse Through Splash
Source: Front Microbiol. 2020 Jul 17;11:1485. doi: 10.3389/fmicb.2020.01485 (PMC7380079; doi:10.3389/fmicb.2020.01485)

# Supplementary Figure S1

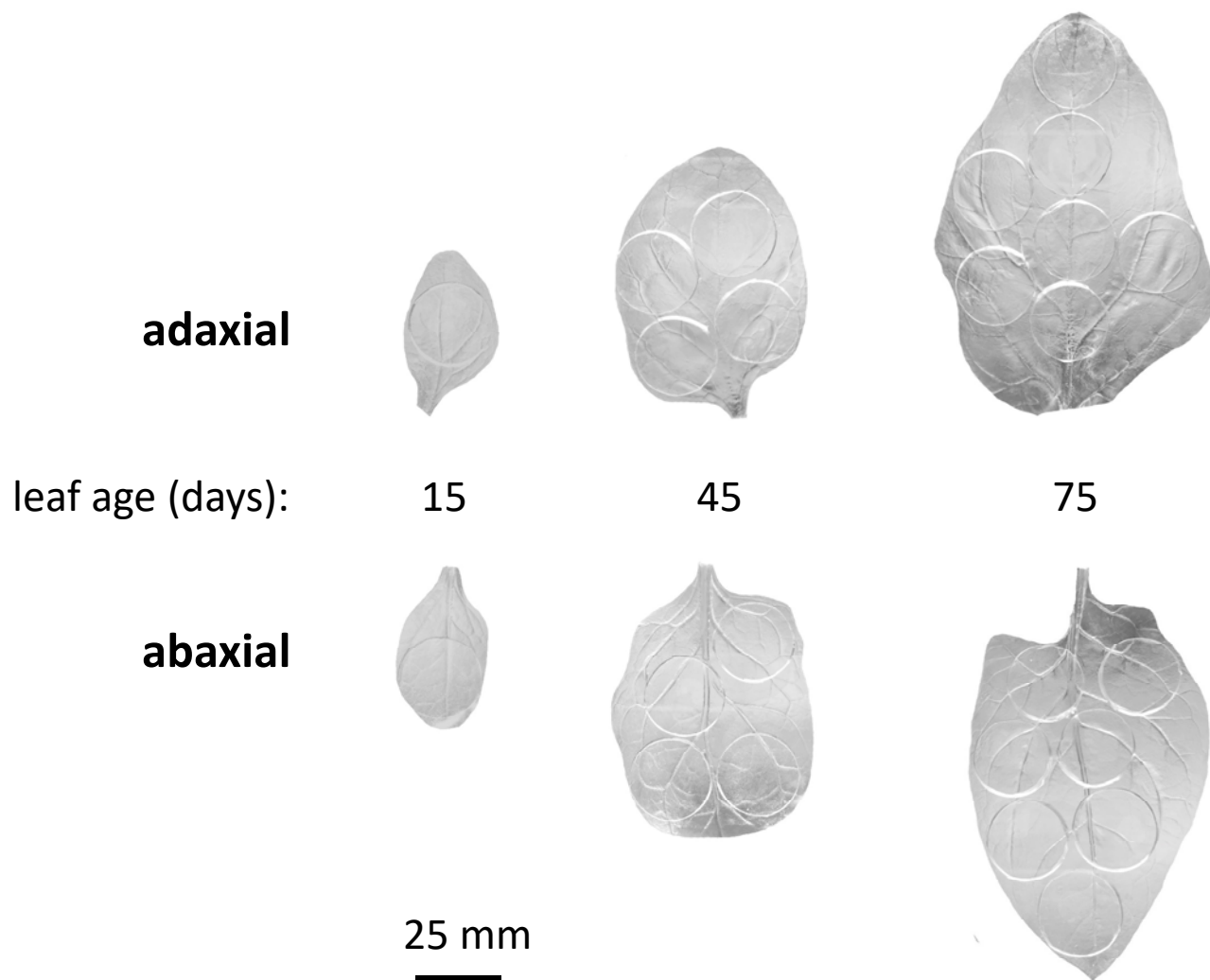

## Supplementary Figure S2

**A**

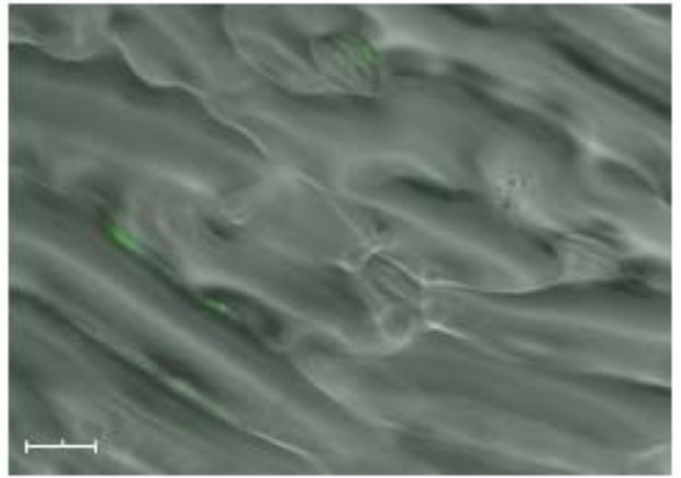

**B**

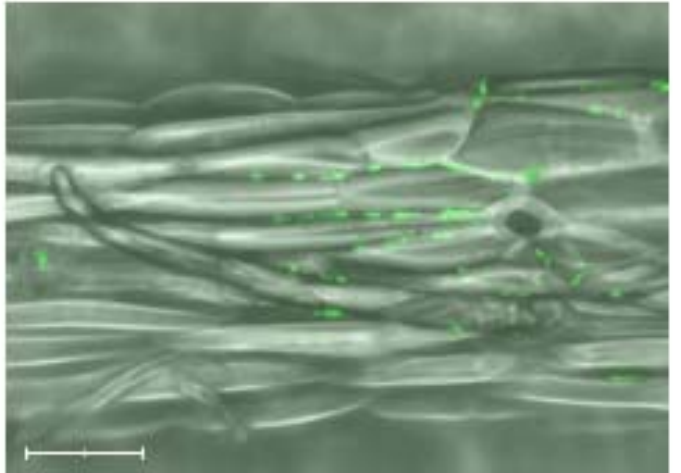

**C**

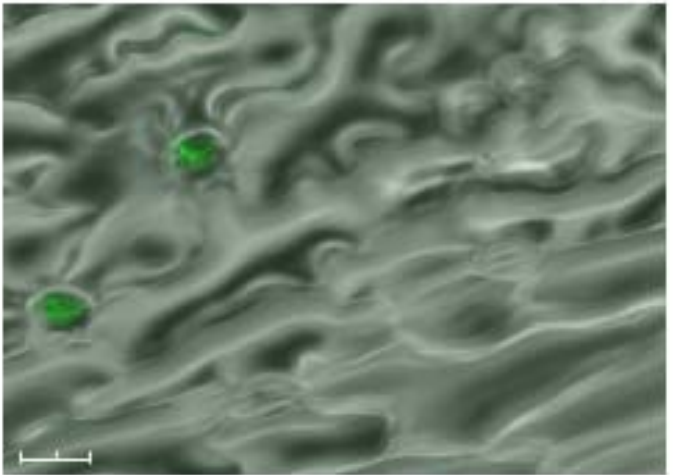

**D**

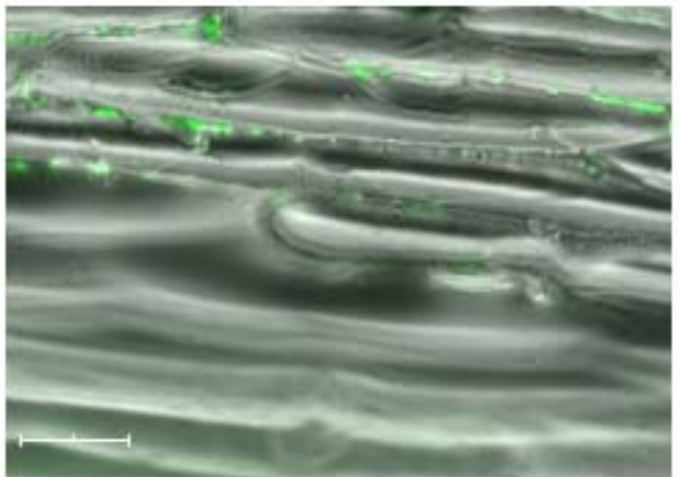

Supplement: FIGURE S1 — Representative PDMS replicasts of abaxial and adaxial surfaces of spinach leaves of different ages. Each one of the replicasts is shown with the coupons that were cut from it using a 24.3 mm diameter corkborer (for the purpose of this figure, coupons were placed back into the cut-out). [file Data_Sheet_1.pdf]
